# A Comprehensive Comparison of the Efficacy and Tolerability of Racecadotril with Other Treatments of Acute Diarrhea in Adults

**DOI:** 10.3389/fmed.2016.00044

**Published:** 2016-10-14

**Authors:** Wolfgang Fischbach, Viola Andresen, Marion Eberlin, Tobias Mueck, Peter Layer

**Affiliations:** ^1^Medizinische Klinik II, Klinikum Aschaffenburg-Alzenau, Aschaffenburg, Germany; ^2^Medizinische Klinik, Israelitisches Krankenhaus, Hamburg, Germany; ^3^Department of Medical Affairs CHC Germany, Boehringer Ingelheim Pharma GmbH & Co KG, Ingelheim, Germany

**Keywords:** diarrhea, racecadotril, loperamide, *Saccharomyces boulardii*, octreotide

## Abstract

Racecadotril is a guideline-recommended treatment to alleviate symptoms of acute diarrhea. A systematic review of randomized studies was performed comparing efficacy and safety of treatment with racecadotril to that with placebo or active treatments in adults. In five double-blind studies, racecadotril and placebo had comparable tolerability, but racecadotril was more effective. This was consistent across multiple efficacy parameters including duration of diarrhea, number of diarrheic stools, abdominal pain, and meteorism; it was also consistent across countries in Africa, Asia, and Europe. In six randomized studies in outpatients comparing racecadotril to loperamide, resolution of symptoms occurred with similar speed and efficacy; however, racecadotril treatment was associated with less rebound constipation and less abdominal discomfort. The seventh comparative study performed in geriatric nursing home residents reported a superior efficacy of racecadotril. In direct comparison with *Saccharomyces boulardii* treatment, racecadotril exhibited similar tolerability but was more efficacious. One study compared racecadotril to octreotide in patients with acute diarrhea requiring hospitalization, rehydration, and antibiotic treatment; in this cohort, octreotide was more efficacious than racecadotril. In conclusion, in adults with acute diarrhea, racecadotril is more efficacious than placebo or *S. boulardii*, similarly efficacious as loperamide and, in patients with moderate to severe disease as add-on to antibiotics, less than octreotide. The tolerability of racecadotril is similar to that of placebo or *S. boulardii* and better than that of loperamide, particularly with regard to risk of rebound constipation. Taken together, these data demonstrate that racecadotril is a suitable treatment to alleviate symptoms of acute diarrhea in adults.

## Introduction

Acute diarrhea causes millions of deaths each year. Most of these occur in children in developing countries, particularly in infants. However, diarrhea is also a significant medical problem in adults and in industrialized countries (1), where it continues to be an important cause of morbidity, health-care utilization, and lost working days (2). For instance, an estimated 375 million episodes of acute diarrhea occur annually in the US, leading to 900,000 hospitalizations and causing 6,000 deaths (3, 4). Infectious enteritis with mandatory reporting to competent authorities also remains high in other industrialized countries such as Germany (5). Infections with bacteria, viruses, and parasites are the most important cause of acute diarrhea; while bacteria are the leading cause of acute diarrhea in the developing countries, viruses are its most frequent cause in industrialized countries (2). Transmission occurs in most cases *via* contaminated water or foodborne (6). Widespread use of broad-spectrum antibiotics and increased foreign travel may further increase the incidence of acute diarrhea in adults from industrialized countries.

As dehydration is the most frequent cause of death in acute diarrhea, oral rehydration therapy is the most important component of treatment. Its increasing use has been associated with a major reduction in deaths due to acute diarrhea (2). While infectious acute diarrhea tends to be self-limiting in otherwise healthy people, it is not only unpleasant but also has societal impact such as lost working days (3). Moreover, even in adults in industrialized countries, diarrhea may lead to death due to visceral failure secondary to dehydration, particularly in the elderly (4).

Therefore, several medications have been developed to alleviate diarrhea symptoms and fasten time to resolution, among which loperamide is used most often. Loperamide is a peripherally acting μ-opiate receptor agonist that has extensively been studied in the treatment of acute diarrhea and is on the List of Essential Medications of the World Health Organization. While clearly effective, loperamide has a number of limitations. First, use of loperamide in the treatment of diarrhea can lead to secondary constipation (7). Second, such constipation may lead to risk of bacterial retention, which is undesirable with toxin-producing bacteria strains (8). Therefore, the US Food and Drug Administration considers loperamide contraindicated in patients with bacterial enterocolitis caused by invasive microorganisms including *Salmonella, Shigella*, and *Campylobacter* species and those with pseudomembranous colitis associated with use of broad-spectrum antibiotics (9). Third, loperamide has a considerable potential for drug–drug interactions as it is metabolized by cytochrome P450 (CYP) 2C8 and 3A4. Accordingly, concomitant use of drugs inhibiting these enzymes can markedly increase loperamide plasma concentrations (9). Moreover, the limited central effects of loperamide are largely driven by it being a substrate for P-glycoprotein. Therefore, P-glycoprotein inhibitors can not only increase loperamide plasma levels (9) but also enhance its access to the brain (10). Based on these drug–drug interactions and also on cases of overdosing and abuse, the US Food and Drug Administration has recently warned about a risk of serious heart problems when using loperamide (11). While loperamide is generally deemed sufficiently safe for self-medication (2), pharmacy customers may insufficiently understand the relevance of such interactions and heed corresponding advice from the pharmacist or package insert.

Probiotics, particularly *Lactobacillus rhamnosus* GG and *Saccharomyces boulardii*, have also repeatedly been studied for the treatment of acute infectious diarrhea. While several guidelines propagate their use, particularly in children, the National Institute for Health and Clinical Excellence in the UK and the Center for Disease Control in the US have concluded that they are not recommended in this indication (12). While the UK National Institute for Health and Clinical Excellence evaluation has acknowledged the superiority of some probiotics relative to placebo in the treatment of acute diarrhea in children, it did not recommend their use because of limitations in the methodology of the underlying studies (13). The US Center for Disease Control mainly criticized that available studies had small sample sizes, raising the possibility that similarly small studies with negative outcomes may not have been reported (14). Only little placebo-controlled data are available evaluating the efficacy of probiotics in the treatment of acute diarrhea in adults. Similarly, various plant extracts based on traditional medicines have been tested in animal models of acute diarrhea, for instance, of *Calea zacatechichi* (15), but little evidence from controlled clinical studies in adults exists.

Somatostatin decreases gastrointestinal motility and intestinal fluid and electrolyte transport but has a short half-life and exhibits tachyphylaxis. Analogs of somatostatin, such as octreotide, may have a longer duration of action. Somatostatin and octreotide regulate mediators of diarrhea at the cellular level and have been tested with favorable results in some types of diarrhea, mostly in patients with AIDS or undergoing cancer chemotherapy (16). However, neither somatostatin nor octreotide has been approved for the indication of acute diarrhea in any major country.

Racecadotril, also known as acetorphan, is an alternative medical option for the treatment of acute diarrhea (17–19). Racecadotril is a low potency inhibitor of neutral endopeptidase (NEP; EC 3.4.24.11, also known as enkephalinase). However, racecadotril is rapidly converted to thiorphan *in vitro* and *in vivo*, which is a much more potent NEP inhibitor (20). Thiorphan has two stereoisomers, the S-enantiomer being referred to as ecadotril or sinorphan and the R-isomer as retorphan or dexecadotril (21). Ecadotril may be somewhat more potent than retorphan, but the difference appears small as compared to the prodrug racecadotril and its alternative metabolite acetyl-thiorphan. Recently, we have comprehensively reviewed the pharmacological profile of racecadotril, including its pharmacokinetics (22). Related to diarrhea treatment, the most important effect of racecadotril is inhibition of the degradation of enkephalins, which in turn have potent antisecretory activity but only little effect on motility in the gut (23). While racecadotril did not inhibit basal secretion in canine jejunum, it inhibited cholera toxin-induced secretion (24). It also inhibited cholera toxin-induced secretion in human jejunum (25) and rotavirus-induced secretion in Caco-2 cells (26). However, in contrast to loperamide, racecadotril did not affect gastrointestinal transit time in rats or mice (27) or in healthy human volunteers (28, 29). Accordingly, loperamide increased *Escherichia coli* content in proximal jejunum and decreased it in stool in newborn piglets, whereas racecadotril did not alter content of the infectious agent in jejunum or stool (30). Based on this mechanism of action, racecadotril has proven effective in castor oil-induced diarrhea in rats (27) and in healthy human volunteers (31). Accordingly, racecadotril is a guideline-recommended treatment of acute diarrhea (2, 32, 33).

Against this background, we have summarized placebo-controlled studies of racecadotril in the treatment of acute diarrhea followed by a comprehensive discussion of direct comparative studies between racecadotril and other medical options in the treatment of acute diarrhea in adult patients.

## Methods

Our analysis is primarily based on dedicated literature searches performed in November 2015 in PubMed and Scopus for the key word combination “racecadotril” and “diarrhea.” Studies were included if they reported direct comparative data of racecadotril and placebo or other treatments of diarrhea in adults; studies in children were not considered. Reference lists of retrieved articles were analyzed for additional publications. There were no language limitations of the search; articles published in English, French, or German were directly analyzed by the authors, one publication in Portuguese was translated into English by a professional translator, and one in Chinese was extracted by a native speaker colleague.

**Figure 1:**
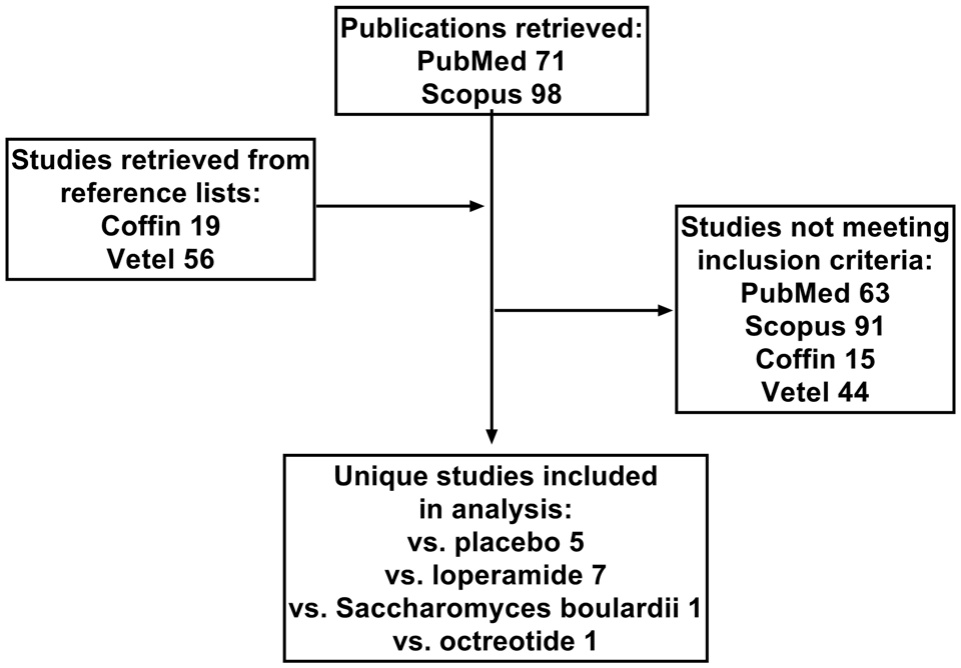
**Flow chart depicting selection of studies included in analysis**. In addition to the two databases, PubMed and Scopus, reference lists from two reviews by Coffin et al. (34) and Vetel et al. (35) were extracted. Numbers indicate number of studies from a given source or with the indicated comparator treatment.

While our manuscript was in preparation, we became aware of an individual patient-based meta-analysis of four placebo-controlled studies (34) and a systematic review and meta-analysis on the efficacy of racecadotril in the treatment of acute diarrhea in adults (35). Both provide important insight into the efficacy of racecadotril in adults with acute diarrhea, and their reference lists were scanned to retrieve studies not listed in PubMed or Scopus (Figure 1). However, we decided to carry on with the present project for two reasons. First, particularly based on the availability of racecadotril as over-the-counter medication in many countries, we feel that a combined analysis of efficacy and tolerability of racecadotril, particularly in comparison to other treatment options, is required for a clinically meaningful understanding of the different profile of treatment options. Second, Vetel et al. have focused on treatment efficacy defined as duration of diarrhea, i.e., time from treatment onset to last unformed stool. While most studies have reported on duration of diarrhea, this has not always been the primary endpoint (36). Moreover, other efficacy parameters, such as meteorism, pain, and nausea, may also be relevant to patients but have not been covered in the analysis by Vetel et al. (35). However, similar to Vetel et al., we have defined studies as out of scope which relate to diarrhea associated with cancer chemotherapy (37–41), cholera (42), or AIDS (43, 44), as these may have different pathologies and hence treatment responsiveness as compared with acute diarrhea. Moreover, studies comparing multiple doses and/or formulations of racecadotril but not including a placebo or active treatment comparator arm have also been considered as out of scope. The flowchart in Figure 1 depicts how studies were selected for inclusion in the present analysis; those studies are listed in Table 1. The present article concomitantly describes error bars as SE or as SD, depending on the choice of the investigators in their original reports.

**Table 1:** **Studies included in the present analysis with corresponding sample size**.

Comparator	Reference	Sample size per arm	Reported efficacy parameters
**Placebo-controlled studies on racecadotril**			
Placebo	Vetel et al. (45)	54–59	Duration of diarrhea (P); number and appearance of stools; percentage of patients cured; concomitant symptoms
Placebo	Baumer et al. (31)	96–102	Duration of diarrhea (P); percentage of patients cured; Kaplan–Meier analysis of presence of diarrhea; reduction of anal burning, spontaneous abdominal pain, nausea, weight loss, pain on abdominal palpation, and meteorism; global efficacy
Placebo	Hamza et al. (36)	32–38	Stool weight on first day of treatment; number of loose stools; pain on abdominal palpation; anal burning; painful anal contraction; spontaneous abdominal pain; abdominal distension; nausea; vomiting; loss of appetite
Placebo	Coffin and Rampal (46)	86–87	Number of diarrheic stools until day 5 (P) and on first day of treatment; duration of diarrhea; associated symptom index; well-being index; global index
Standard treatment	Yao and Xi (47)	54–55	Duration of diarrhea (P); treatment duration; percentage of patients cured
**Actively controlled studies on racecadotril**			
Loperamide	Roge et al. (48)	32–37	Duration of diarrhea (P); cumulative recovery on day 2 (P); abdominal pain; abdominal distension
Loperamide	Vetel et al. (49)	75–82	Number of stools until recovery (P); time to cure; physician’s global evaluation; asthenia; abdominal distension; anorexia; pain on abdominal palpation; spontaneous abdominal pain; nausea; anal burning; vomiting
Loperamide	Prado and Global Adult Racecadotril Study Group (50)	472–473	Duration of diarrhea (P); recovery rate after 72 h; overall clinical success; duration of abdominal pain and of abdominal distension
Loperamide	Wang et al. (51)	31	Duration of diarrhea, abdominal pain, and abdominal distension; improvement rate of anal burn and nausea; physician-determined overall clinical success
Loperamide	Hu and Sun (52)	111–112	Duration of diarrhea in Kaplan–Meier analysis (P); resolution rate at 24, 48, and 72 h; percentage of patients reporting resolution of spontaneous abdominal pain, pain on abdominal palpation, abdominal distension, anorexia, nausea, and anal burning
Loperamide	Coulden et al. (53)	60	Duration of diarrhea (P); time to resolution of spontaneous abdominal pain and abdominal distension; prevalence of spontaneous abdominal pain, pain on abdominal palpation, abdominal distension, anorexia, nausea, and anal burning
Loperamide	Galleli et al. (54)	30–31	Number of diarrhea episodes; duration of abdominal pain and diarrhea; stool weight until recovery
*Saccharomyces boulardii*	Moraes et al. (55)	161–175	Clinical success as judged by investigator; duration of diarrhea; number of bowel movements until recovery; prevalence of spontaneous abdominal pain, pain on abdominal palpation, abdominal distension, anorexia, nausea, and anal burning on day 2; Kaplan–Meier analysis of probability of cure
Octreotide	Mehta et al. (56)	50	Daily number of stools until recovery (P); daily quantity of stools; required volume of fluid substitution

*See main text and references for details*.

*(P), primary endpoint; where not stated, no efficacy parameter has been reported as primary endpoint*.

## Placebo-Controlled Studies on Racecadotril in Adults

Five double-blind, randomized studies have explored the efficacy and tolerability of racecadotril relative to placebo in acute diarrhea in adults. An initial multi-center dose-ranging study performed in France compared racecadotril capsules of 30, 100, and 300 mg to placebo, each administered thrice daily prior to a meal (45). The main inclusion criterion was diarrhea lasting not more than 5 days (mean 1.5 days) with at least three unformed stools within the past 24 h (mean 6). Key exclusion criteria were chronic diarrhea of any cause, other complaints that may cause repeated or chronic diarrhea, alternating bouts of diarrhea and constipation, iatrogenic diarrhea, and concomitant diseases that might affect vital signs. Treatment lasted until the occurrence of the first formed stool or a period of 12 h without any stools but could not exceed 10 days. The primary efficacy endpoint was time to cure; secondary efficacy endpoints were self-assessed number and appearance of stools, percentage of patients cured and concomitant symptoms during consultations. Safety endpoints were based on clinical examination, reported side effects, and standard laboratory tests. Samples sizes were 49–55 per group, and most patients had negative stool cultures. The mean times to disappearance of loose or liquid stools were 72.0, 68.4, 69.6, and 65.0 h in the placebo, 30, 100, and 300 mg racecadotril groups, respectively (not significantly different). Cure rates were 90–93% across all treatment groups. Three adverse events were reported in the placebo group (3/49; headache, nausea, cystitis) and 5 in the combined racecadotril groups [5/164; nausea (2 patients), meteorism, dizziness, and bad taste]. Number of loose stools was 2.7 vs. 2.1 (*P* = 0.06) in the first 9 h and 8.6 vs. 7.1 (*P* = 0.03) in the first 60 h with no evident dose-dependency among the racecadotril groups. Based on these findings, all subsequent placebo-controlled studies have focused on the 100 mg dose of racecadotril.

Another multi-center, double-blind study also performed in France has used comparable inclusion and exclusion criteria and randomized 198 patients (18–89 years) to receive 100 mg racecadotril or placebo, two capsules at start of treatment and one after each unformed stool, until resolution of diarrhea (31). Mean duration of diarrhea prior to start of treatment was 1.7 and 1.6 days, and mean number of loose stools in the preceding 24 h was 5.3 and 4.9, respectively. Five patients, all in the placebo group, prematurely discontinued the study because diarrhea worsened or failed to improve. Moreover, at study-end visit (10–14 days after initiation of treatment), 30 patients reported the continued presence of unformed stools. These 35 patients were excluded from the analysis of the primary endpoint, time to cure (7.4 vs. 23.5% in the racecadotril and placebo group, respectively). The remaining patients reported a duration of diarrhea during treatment of 3.4 ± 0.1 days in the racecadotril and 4.4 ± 0.2 days in the placebo group (*P* = 0.001). As fewer patients on racecadotril failed to achieve cure during the observation period, the difference between treatments would have been larger in an intention-to-treat analysis of all participants. Accordingly, a Kaplan–Meier type of analysis for probability of presence of diarrhea, including also the 35 patients without cure, also confirmed superiority of racecadotril over placebo (*P* < 0.001). Thus, the probability of reporting cure on day 4 was 75 ± 5% in the racecadotril as compared with 37 ± 5% in the placebo group. As compared to placebo, racecadotril also caused a significantly greater reduction of anal burning, spontaneous abdominal pain, nausea, weight loss, pain on abdominal palpation, and meteorism. Global efficacy rating by the physician on a visual analog scale of 1–100 was 83 ± 2 vs. 61 ± 3, respectively, and by the patient 82 ± 2 vs. 62 ± 3, respectively (*P* < 0.001). Approximately 16 (16.8%) vs. 18 (18.4%) of patients on racecadotril as compared to placebo reported a total number of 35 vs. 36 adverse events (n.s.). These included nausea, thirst, dizziness, constipation, and headache and were largely of mild to moderate intensity in both groups. Tolerability rating on a visual analog scale by physicians was 89 ± 2 vs. 89 ± 1 and by patients 93 ± 2 vs. 87 ± 2 (n.s.).

The third double-blind study in adults with acute diarrhea performed in Tunisia has used similar key inclusion and exclusion criteria, and randomized 71 patients to receive 100 mg racecadotril or placebo thrice daily until diarrhea had ended for a maximum of six treatment days (36). In contrast to the above studies, the primary efficacy endpoint was accumulated stool weight during the first day of treatment. Causative microorganisms were identified in only five patients. Mean stool weight during the first day of treatment was 355 ± 35 g with racecadotril treatment as compared to 499 ± 46 g with placebo treatment (*P* = 0.025), corresponding to a 28.9% reduction in stool weight by racecadotril treatment. Number of loose stools in the 24 h prior to start of treatment was 6.4 ± 0.5 vs. 6.3 ± 0.4, and after 1 day of treatment this had declined to 4.3 ± 0.4 vs. 5.4 ± 0.4 in the racecadotril and placebo groups, respectively (*P* = 0.027). On day 2, 15.6% of patients receiving racecadotril passed at least one formed stool as compared to 5.3% in the placebo group. While pain on abdominal palpation on day 4 was similar in both groups (10.7 vs. 9.7%), patients receiving racecadotril reported less anal burning (18.2 vs. 25%), painful anal contraction (0 vs. 12.5%), spontaneous abdominal pain (22.6 vs. 27.3%), abdominal distension (5.6 vs. 18.2%), nausea (4.8 vs. 16.7%), vomiting (0 vs. 12.5%), and loss of appetite (15.4 vs. 18.8%). Adverse events were reported by 3.1 and 5.3% in the racecadotril and placebo group. Physician’s global tolerability assessment on a 1–100 scale was 96.1 ± 4.2 vs. 94.2 ± 16.5, respectively.

The fourth double-blind, double dummy study performed in France in adults with acute diarrhea has also used similar key inclusion and exclusion criteria except that maximum prior duration of diarrhea was limited to 3 days (46). This study has randomized 259 patients to receive one of the three treatments: racecadotril 100 mg capsules, dexecadotril 75 mg tablets, or placebo thrice daily until recovery (12 h without stools or two consecutive normal stools) for a maximum of 5 days. Of note, the primary analysis of this study compared dexecadotril to either racecadotril or placebo; therefore, no statistical analysis of the comparison of efficacy of racecadotril and placebo was reported. A total of 22 patients prematurely discontinued the study: 6 in the racecadotril group (3 due to lack of efficacy, 3 due to unauthorized concomitant treatment before or during study), 6 in the dexecadotril group (4 due to lack of efficacy, 2 due to adverse events), and 10 in the placebo group (5 due to lack of efficacy, 2 each due to concomitant disease and loss to follow-up, 1 due to poor compliance). The median number of diarrheic stools in the 24 h prior to start of treatment was 5–6. The primary efficacy endpoint was number of diarrheic stools from start of treatment until recovery or day 5; this was 5, 3, and 9 with racecadotril, dexecadotril, and placebo, respectively, in the intention-to-treat analysis. Upon adjustment for stool quality (normal = 0, loose = 1, watery = 2), median weighted number was 8, 4 and 15, respectively. Among the secondary endpoints, median duration of diarrhea was 30, 13, and 67 h, respectively. On day 5, 96.5, 94.2, and 82.8% of patients had recovered, respectively. The symptom indices were used as secondary efficacy endpoints. An associated symptom index consisting of spontaneous abdominal pain, bloating, gurgling, nausea, vomiting, loss of appetite, fever, and asthenia; each symptom was weighted by intensity from 0 (none) to 3 (important). A well-being index based on impairment of daily activities, diet, sleep, and feeling of discomfort; each of the four was weighted from 0 (none) to 3 (severe). A global index was calculated as the arithmetic mean of the associated symptom index, the well-being index, and the number of diarrheic stools divided by 3. While the associated symptom index and the well-being index were comparable across groups at baseline (1.0–1.3 and 1.2–1.3), the global index differed between groups with 10, 10.7, and 9.2 in patients to receive racecadotril, dexecadotril, and placebo, respectively. At study end, the associated symptom index was 1.7, 1.6, and 2.3, the well-being index 1.8, 1.4, and 2.3, and the global index 11.3, 8.8, and 17.8 in the racecadotril, dexecadotril, and placebo groups, respectively. A total of 25 patients reported a total of 34 adverse events during the study; this included 9 in the racecadotril, 14 in the dexecadotril, and 11 in the placebo group. Most of them were of mild to moderate intensity and had disappeared at study end.

These four placebo-controlled studies have been subject to two meta-analyses. One of them focused on the duration of diarrhea as efficacy and constipation as safety endpoint; it had also included a study in which the comparator arm was treated with the probiotic *S. boulardii* (35). In this analysis, the hazard ratio for a greater efficacy with racecadotril was 1.65 (confidence interval: 1.38–1.97). In contrast, the hazard ratio for safety was 0.95 (0.24–3.68). The second meta-analysis included only the four placebo-controlled studies, was based on individual patient raw data, and included a wider range of efficacy endpoints (34). In contrast to some of the primary study reports, this analysis included all randomized patients on an intention-to-treat based, i.e., 669 patients. This included 59 and 56 receiving a 30- or 300-mg dose, respectively, from the dose-ranging study (45), leaving 282 patients receiving placebo and 272 patients receiving 100 mg racecadotril. At baseline, the latter two groups were well balanced with regard to age (41 years), gender (53.5% females), weight (66.6 kg), height (1.68 m), ethnicity (17.9% non-Caucasian), blood in stool (3.1%), watery stool aspect (91.0%), work interruption (41.0%), number of stools in last 24 h prior to treatment start (5.72), and additional symptoms including anal burning (57.2%), anal contractures (44.7%), spontaneous abdominal pain (91.6%), nausea (64.8%), vomiting (26.6%), appetite loss (77.7%), fatigue (82.7%), insomnia (50.0%), pain on palpitation (84.3%), and meteorism (76.2%). The primary efficacy parameter of the meta-analysis was duration of diarrhea. In a model adjusting for age, weight, and clinical global impression, the 100 mg racecadotril dose (identified to be the minimally effective dose) as compared to placebo exhibited a hazard ratio for benefit of 1.85 [1.54; 2.23]. For analysis of overall symptom score, pain and nausea defined as number of symptoms on day 3 of treatment, the decrease from baseline depended on baseline severity and clinical global impression. When adjusting for these baseline variables, the hazard ratio for having the respective symptom with 100 mg racecadotril as compared to placebo was 0.73 [0.66; 0.80] for overall symptom score, 0.41 [0.30; 0.56] for nausea and vomiting and 0.52 [0.42; 0.65] for abdominal pain. For responder analysis (recovery within 3 days after baseline), the hazard ratio was 1.602 [1.365; 1.881], for number of diarrheic stools 0.743 [0.650; 0.849], and for sick leave duration 0.666 [0.528; 0.840].

**Figure 2:**
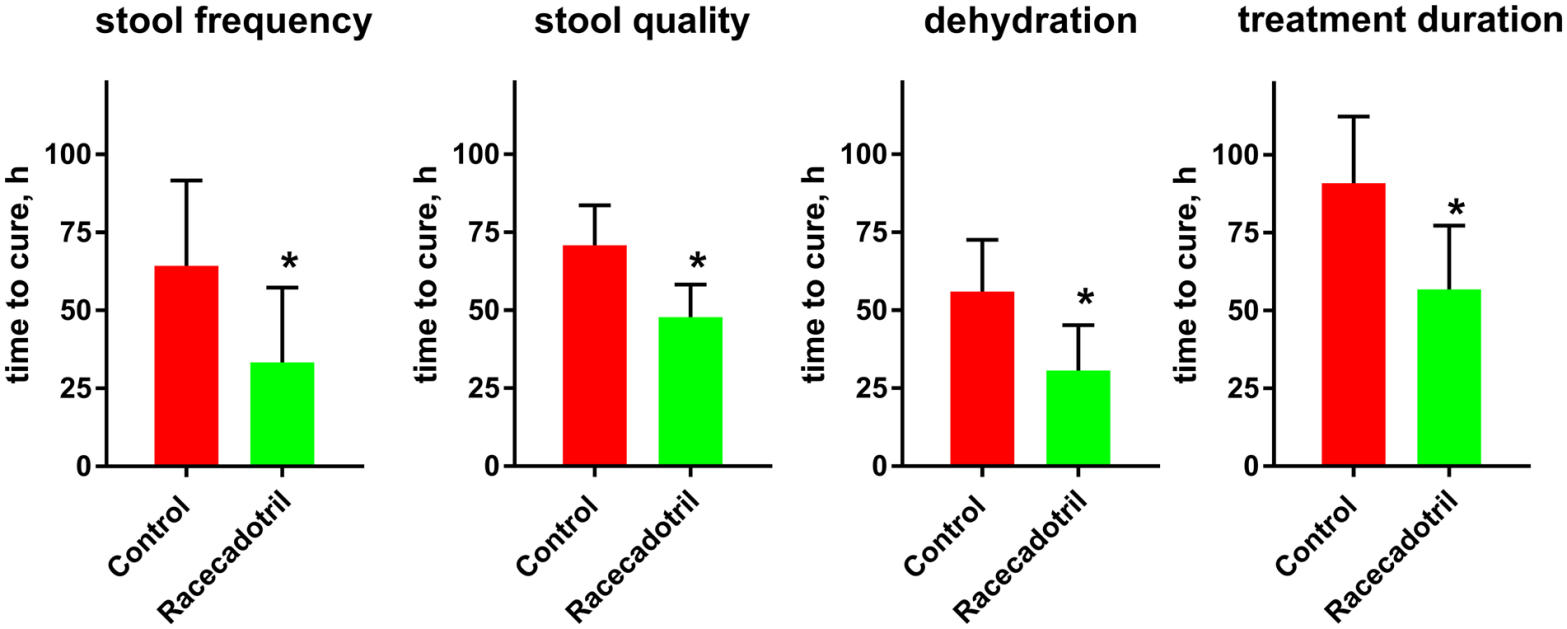
**Comparison of time to cure between rehydration treatment (“control”; *n* = 54) and rehydration treatment plus racecadotril (“racecadotril”; *n* = 55) in a randomized study**. Of note, cure was observed in 39/54 patients (70.2%) in the control and 50/55 patients (90.9%) in the racecadotril group after 72 h of treatment. Data are means ± SD. *P < 0.05 vs. control. Drawn based on data published in Yao and Xi (47).

The fifth study performed in China and not included in any of the previous meta-analyses or other reviews, randomized 109 adults with acute diarrhea to receive standardized oral rehydration therapy or rehydration plus 100 mg racecadotril or placebo thrice daily (47). Prior to treatment, diarrhea existed for a mean of 34 h with a mean frequency of about 7.4 times per day; vomiting and dehydration/electrolyte disturbances existed in 33–36% of patients. Time to cure was the primary endpoint; this was 64.3 ± 27.3 h in the control and 33.3 ± 24.0 h in the racecadotril group when defined based on frequency of stools, 70.8 ± 12.8 vs. 47.8 ± 10.5 h when defined based on quality of stools and 56.0 ± 16.6 vs. 30.7 ± 14.5 h when defined based on presence of dehydration/electrolyte disturbance (Figure 2). Accordingly, treatment duration was longer in the control than in the racecadotril group (90.9 ± 21.4 vs. 56.8 ± 20.5 h). Seventy-two hours after start of treatment, cure was observed 39/54 patients in the control and 50/55 patients in the racecadotril group (70.2 vs. 90.9%).

In summary, the above individual placebo-controlled studies as well as the literature-based (35) and the individual patient-based meta-analyses (34) demonstrate that racecadotril is superior to placebo in the treatment of acute diarrhea in adults. This was consistent across multiple efficacy parameters including duration of diarrhea, number of diarrheic stools, abdominal pain, and meteorism (Table 2). Moreover, it was consistent across studies performed in France, Tunisia, and China, likely to represent different patient populations. From a socio-economic point of view, a shortening of sick leave with racecadotril as compared to placebo is noteworthy. On the other hand, the tolerability was similar to that observed with placebo and, specifically, no rebound constipation was observed.

**Table 2:** **Effect of racecadotril as compared to placebo or active treatment for the secondary symptoms meteorism/abdominal distension and abdominal pain**.

Comparator	Reference	Meteorism/abdominal tension/bloating		Spontaneous abdominal pain	
		Control	Racecadotril	Control	Racecadotril
**Placebo-controlled studies on racecadotril**					
Placebo	Vetel et al. (45)	n.r.	n.r.	n.r.	n.r.
Placebo	Baumer et al. (31)	34.7%	18.3%*	20.5%	9.6%*
Placebo	Hamza et al. (36)	18.2%	5.6%	27.3%	22.6%
Placebo	Coffin and Rampal (46)	n.r.	n.r.	n.r.	n.r.
Standard treatment	Yao and Xi (47)	n.r.	n.r.	n.r.	n.r.
**Actively controlled studies on racecadotril**					
Loperamide	Roge et al. (48)	50.0%	27.0%*	59.4%	40.5%*
Loperamide	Vetel et al. (49)	Comparable		Comparable	
Loperamide	Prado and Global Adult Racecadotril Study Group (50)	24.4 h	5.4 h*	11.0 h	10.0 h
Loperamide	Wang et al. (51)	12 h	12 h	14 h	16 h
Loperamide	Galleli et al. (54)	n.r.	n.r.	28 h	14 h*
Loperamide	Hu and Sun (52)	87%	88%	85%	91%
Loperamide	Coulden et al. (53)	2 days	1 day	1 day	1 day
*Saccharomyces boulardii*	Moraes et al. (55)	6.21%	6.29%	12.42%	6.86%
Octreotide	Mehta et al. (56)	n.r.	n.r.	n.r.	n.r.

Data are percentage of patients reporting a given symptom during treatment or time (hours or days presented as mean or median) until resolution. Note that symptom assessment during treatment was performed on different treatment days in the various studies; moreover, some studies reported graphical rather than quantitative data (49). n.r., not reported; *P < 0.05 vs. control. In an individual patient-based meta-analysis, the hazard ratio for reporting abdominal pain with racecadotril vs. placebo was 0.53 [0.34; 0.82] (34).

## Actively Controlled Studies in Adults

Our search has identified nine studies that compared racecadotril to an active control in the treatment of acute diarrhea in adults. Seven of them compared racecadotril to loperamide, one to the probiotic *S. boulardii*, and one to the somatostatin analog octreotide. The first double-blind study, performed in France, randomized 69 patients to receive either racecadotril (100 mg; *n* = 37) or loperamide (1.33 mg; *n* = 32); in either case, two capsules were given at start of treatment, another two 12 h later, and then one capsule thrice daily until recovery for a maximum of 7 days (48). Key inclusion and exclusion criteria were similar to the above placebo-controlled studies. The main efficacy parameters were time to resolution (2.2 vs. 2.3 days for racecadotril and loperamide) and cumulative recovery on day 2 (59.3 vs. 50.0%; n.s. for both parameters). The percentage of patients reporting abdominal pain for more than 1 day also did not differ significantly between treatments (40.5% with racecadotril vs. 59.4% with loperamide), whereas duration of abdominal distension (1.1 vs. 1.8 days) and percentage of patients reporting abdominal distension for more than 1 day (40.5 vs. 59.4%) was significantly smaller with racecadotril than with loperamide. The only reported adverse event, percentage of patients with constipation after resolution of diarrhea was significantly less with racecadotril than with loperamide (8.1 vs. 31.3%).

The second double-blind study was also performed in France, used similar key inclusion and exclusion criteria and randomized 157 adults with acute diarrhea but used a slightly different treatment design as compared to the Roge et al. (48) study (49). One group of patients received 100 mg racecadotril at start of study, followed by thrice daily 100 mg to be taken before each meal (*n* = 82); the other group received 2 mg of loperamide at start of study, followed by another 2 mg after each diarrheic stool (*n* = 75). Both treatments were continued until recovery for a maximum of 7 days. At inclusion, duration of diarrhea (39.4 vs. 41.4 h) and number of stools during past 24 h (5.9 vs. 5.3) were similar in both groups. Eight patients were withdrawn from the study prior to visit 2 (six in racecadotril group including two due to lack of efficacy, two due to use of non-permitted concomitant medications, one each due to loss to follow-up and withdrawal of consent; two in the loperamide group with one each for lack of efficacy and withdrawal of consent). Moreover, 5 patients in each group failed to fill the evaluation sheets correctly and could not be evaluated for efficacy, leaving a total of 77 patients on racecadotril and 70 on loperamide for evaluation. The primary efficacy endpoint, number of stools passed until recovery, was similar for racecadotril and loperamide (3.5 vs. 2.9). Among secondary efficacy parameters, duration of diarrhea (14.9 vs. 13.7 h) and physician’s global evaluation (83.7 vs. 82.3) were also similar in both groups. Other efficacy parameters, including asthenia, abdominal distension, anorexia, pain on abdominal palpation, spontaneous abdominal pain, nausea, anal burning, and vomiting, also exhibited similar frequency in both groups at study end. The incidence of adverse events was 7.4% with racecadotril and 12% with loperamide, mostly reported as being of mild to moderate intensity in both groups. Rebound constipation, defined as lack of any stool for at least 2 days during treatment and not counted as part of adverse events by the authors, was seen in 9.8% of racecadotril as compared to 18.7% of loperamide patients; among patients reporting constipation, its duration was 1.3 days in the racecadotril vs. 1.6 days in the loperamide group.

The third actively controlled study has used a somewhat different design (50). While key exclusion criteria and maximum pre-existing duration of diarrhea (up to 5 days) were similar to other studies, inclusion required at least three watery stools in the past 24 h. While other controlled studies involved various centers within 1 country, this study recruited patients from 14 countries in Latin America, Africa, and Asia. In a single-blind approach, 945 patients were randomized to receive 100 mg racecadotril (*n* = 473) or 2 mg loperamide (*n* = 472) at start of study and then thrice daily until resolution of diarrhea (no stool for 12 h or two consecutive normal stools) for a maximum of 7 days. Mean duration of diarrhea at inclusion was 2.1 days in both groups and number of watery stools in the past 24 h was 6.4 and 6.5. The primary efficacy parameter was duration of diarrhea, defined as time from first dose of study medication to appearance of first formed stool, and was 55.0 h in both groups. Recovery rate after 72 h and overall clinical success (92 vs. 93%) were also similar in both groups. Duration of abdominal pain (11.0 vs. 10.0 h) was similar in both groups, but duration of abdominal distension was significantly shorter with racecadotril (5.4 vs. 24.4 h). Constipation, defined as at least 36 h without passing a stool, occurred less often with racecadotril than with loperamide treatment (16 vs. 25%). Approximately 108 patients reported at least one adverse event, with 14.2% in the racecadotril and 23.9% in the loperamide group (*P* = 0.001); similarly, adverse events considered related to treatment by the investigator occurred in 9 vs. 18% of patients. Four patients receiving racecadotril (1 related) and 10 receiving loperamide (7 related) reported adverse events rated as severe by the investigator. Specific adverse events occurring significantly less often with racecadotril than with loperamide included constipation (as spontaneously reported 3.4 vs. 12.5%), enlarged abdomen (1.7 vs. 6.1%), anorexia (0.8 vs. 2.3%), and abdominal pain (0.2 vs. 1.9%).

The fourth actively controlled study was performed in Taiwan (51) had similar key inclusion and exclusion criteria and study design as the Prado and Global Adult Racecadotril Study Group (50) study. However, this study randomized 62 patients to single-blind treatment with either 100 mg racecadotril thrice daily or 2 mg loperamide twice daily until recovery for a maximum treatment of 7 days. With the limited sample size of 31 patients per group, duration of diarrhea (19.5 vs. 13.0 h), of abdominal pain (16 vs. 14 h), and of abdominal distension (12 vs. 12 h) and improvement rates of anal burn (71.0 vs. 74.2%) and nausea (74.2 vs. 77.4%) as well as physician-judged overall clinical success (87.1 vs. 87.1%) did not differ significantly between groups in the intention-to-treat analysis. Eight patients on racecadotril and seven on loperamide reported at least one adverse event; however, incidence of constipation was significantly smaller with racecadotril than with loperamide (12.9 vs. 29.0%).

**Figure 3:**
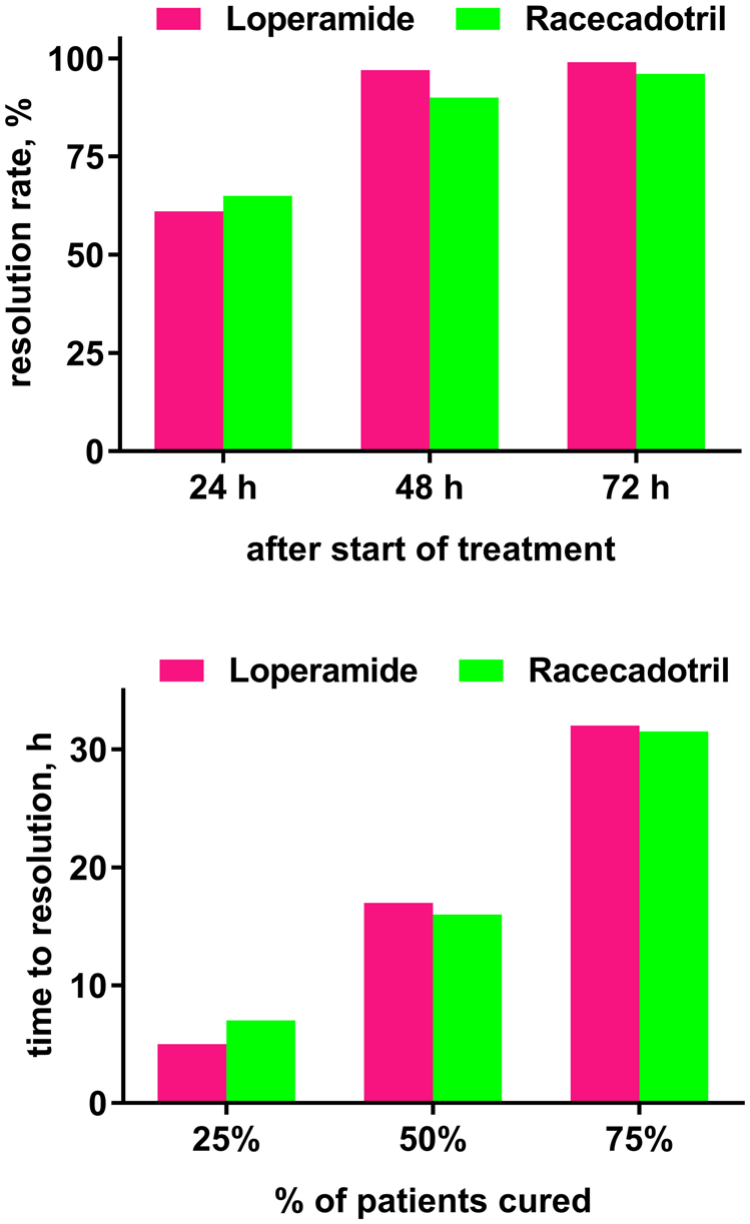
**Time course of efficacy (time to resolution of diarrhea) in a randomized study comparing loperamide (*n* = 111) and racecadotril (*n* = 112) based on the intention-to-treat population**. Data are shown as resolution rates observed at indicated time points (upper panel) and time at which a given percentage of patients reported cure (lower panel). Group differences did not differ significantly. Drawn based on Hu and Sun (52).

The fifth, observer-blinded study, performed in China, randomized 223 adults with acute diarrhea to receive 100 mg racecadotril or 2 mg loperamide thrice daily until recovery, defined as 12 h with no stools or two consecutive normal stools, for a maximum of 3 days (52). Twelve patients in the racecadotril group (eight due to protocol violation, four due to loss of follow-up) and one in the loperamide group (protocol deviation) did not complete the study. The primary endpoint was time to cure and did not differ significantly between both groups in a Kaplan–Meier analysis in the intention-to-treat or the per-protocol analysis. Resolution rates at 24, 48, and 72 h after initiation of treatment were 65 vs. 61%, 90 vs. 97%, and 96 vs. 99% in the intention-to-treat analysis in the racecadotril and loperamide group, respectively (n.s.; Figure 3). The 25% fastest responders had a time to cure of 7 h with racecadotril as compared to 5 h with loperamide, the fastest 50% of 16 vs. 17 h, and the fastest 75% of 32 h in both groups. The percentage of patients reporting resolution of specific symptoms with racecadotril and loperamide in the intention-to-treat analysis did not significantly differ between treatments (91 vs. 85% for spontaneous abdominal pain, 89 vs. 92% for pain on abdominal palpation, 88 vs. 87% for abdominal distension, 93 vs. 87% for anorexia, 98 vs. 100% for nausea, and 96 vs. 100% for anal burning). In the per-protocol analysis, racecadotril was numerically more effective than loperamide for spontaneous abdominal pain, pain on abdominal palpation, abdominal distension, and anorexia (reach statistical significance for the former three symptoms), whereas it was 100% in both groups for nausea and anal burning. No patient in either group withdrew due to adverse events. Adverse events were reported in 4 patients in the racecadotril group (2 with abdominal distension, 1 each with abdominal pain or anorexia) and 24 in the loperamide group (11 with abdominal distension, 4 with abdominal pain, 3 with constipation, 5 with nausea, and 1 with anorexia), of which 4 and 19, respectively, were judged to be treatment-related by the investigator. All adverse events in both groups were classified as being of mild to moderate intensity by the investigator.

The sixth, observer-blinded study, performed in India, randomized 120 adults with acute diarrhea to receive either 100 mg racecadotril or 2 mg loperamide thrice daily until recovery for a maximum of 7 days (53). Five patients in either group withdrew from the study (four with adverse events and one with protocol deviation in the racecadotril group, two each with adverse events or protocol deviation, and one with lack of efficacy in the loperamide group). Eight and six patients in the racecadotril and loperamide group, respectively, had protocol deviations, leaving 52 and 54, respectively, for a per-protocol analysis. The primary endpoint was median duration of diarrhea, which was 3 days in both groups. Among secondary efficacy endpoints, duration to resolution of spontaneous abdominal pain was 1 day each for both treatments and 1 vs. 2 days with racecadotril vs. loperamide for abdominal distension. Both treatments reduced the prevalence of individual symptoms from start of treatment to study end (spontaneous abdominal pain: from 77 to 2% for racecadotril and 72 to 7% for loperamide; pain on abdominal palpation: from 48 to 0% and 48 to 3%; abdominal distension: from 23 to 0% and 28 to 5%; anorexia: from 27 to 3% and 32 to 5%; nausea: 38 to 2% and 37 to 5%; anal burning: from 5 to 0% and from 7 to 2%). Ten patients (16.7%) in the racecadotril (including two with constipation, one each with enlarged abdomen, abdominal pain, fever, or nausea) and eight (13.3%) in the loperamide group (including three with constipation, two with enlarged abdomen, and one each with abdominal pain, fever, or nausea) reported adverse events. Most of them in either group were judged to be of moderate intensity.

The seventh direct comparative trial between racecadotril and loperamide was not performed in an outpatient setting as all other placebo or actively controlled trials but rather in geriatric nursing homes in Italy (54). Sixty-one patients were randomized in a double-blind manner to receive 100 mg racecadotril at treatment start and then every 8 h or 2 mg loperamide twice at treatment start and then after each unformed stool with a maximum of four tables within 24 h. Treatment was started after the third observed diarrhea episode and continued until resolution (two consecutive normal stools or no stool for 12 h) for a maximum of 4 days. While administration of fluid, specifically standardized oral rehydration solution, had not been defined by protocol in the above or actively controlled studies, 750 ml of such solution was administered daily as base treatment in both groups throughout the study. While other studies had involved patients with a mean age of about 40 years, participants in this study were about 82 years of age (range: 73–96). Number of diarrhea episodes after start of treatment was 3.93 with racecadotril as compared to 7.29 with loperamide. In the intention-to-treat population, patients treated with racecadotril experienced a significantly shorter duration of abdominal pain (14 vs. 28 h) and of diarrhea (36 vs. 63 h) and less stool output until recovery (120 vs. 150 g/kg). Four patients in the loperamide group did not exhibit resolution of diarrhea with loperamide, but rapidly recovered after switch to racecadotril. At least one adverse event was reported by 12% of racecadotril and 60% of loperamide patients. This difference was driven by the occurrence of nausea (10 vs. 20%) and constipation (15 vs. 60%), whereas other adverse events did not differ between groups. Patients not responsive to loperamide were genotyped for main allelic variants of CYP 3A4/5 and 2C8; however, neither detrimental alleles nor extra copies of functional alleles were detected, indicating that they were neither ultra-rapid nor poor metabolizers for these two enzymes. In an additional pharmacoeconomic analysis based on the intention-to-treat population, average treatment cost was € 44.85 in the racecadotril and € 91.99 in the loperamide group.

A meta-analysis on the above seven studies has been reported (35). Based on these seven randomized studies with 1618 patients, proportion of recovered patients was similar to racecadotril and loperamide (hazard ratio 1.08 [0.95; 1.22]). However, a significantly lower proportion of patients treated with racecadotril reported constipation as compared to those receiving loperamide (hazard ratio 0.34 [0.22; 0.51]).

**Figure 4:**
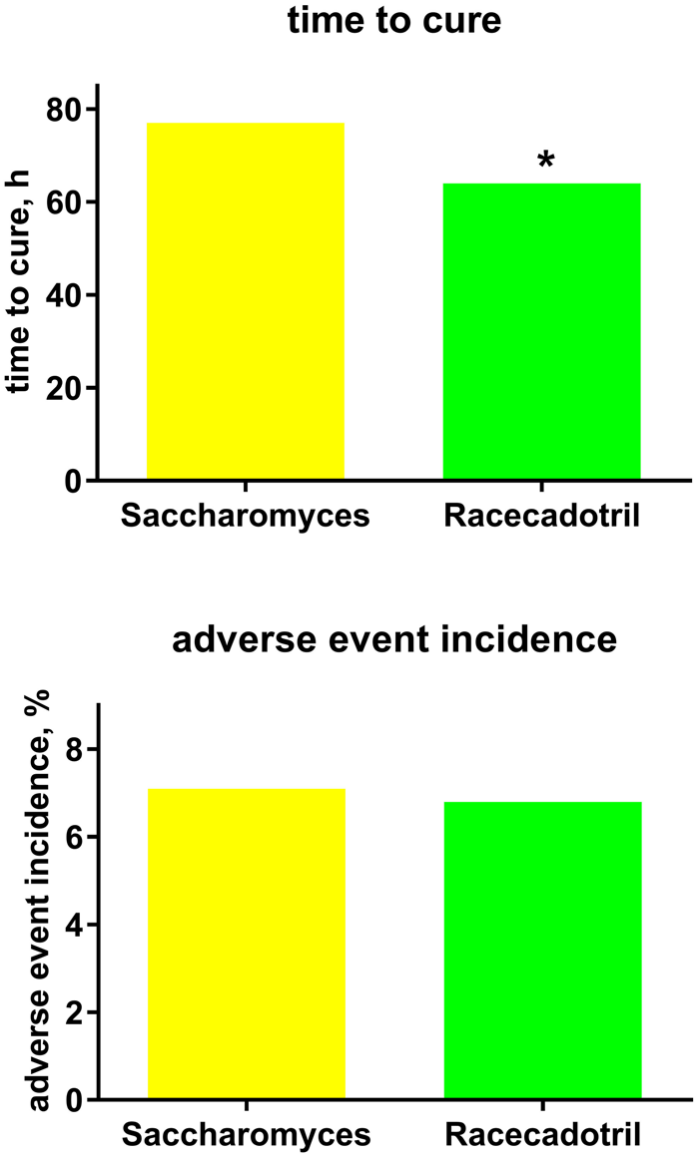
**Treatment efficacy (time to cure) and tolerability (adverse event incidence) of racecadotril in comparison with *Saccharomyces boulardii* [“*Saccharomyces*.”; (55)]**. Data are means of 197 vs. 207 patients. *P < 0.05 vs. *Saccharomyces* with no error bar reported by original authors, and no statistical analysis reported for tolerability.

Our search has identified two actively controlled racecadotril studies in adults with acute diarrhea, which have used an active comparator other than loperamide. One was an investigator-blinded study performed in Brazil which randomized 334 adults to receive either one 100 mg capsule of racecadotril every 8 h (*n* = 175) or two capsules of 100 mg *S. boulardii* (Floratil^®^, Merck; *n* = 161) every 12 h (55). Treatment duration was planned until recovery (two consecutive normal bowel movements or no bowel movement for 12 h) for a maximum of 7 days. Duration of diarrhea prior to start of treatment was 2.1 vs. 2.0 days, and number of bowel movements in the last 24 h prior to treatment was 7.0 in both groups. Clinical success as judged by the investigator was 96.6% with racecadotril as compared to 96.9% with *S. boulardii*. However, time to recovery (64 vs. 77 h; Figure 4) and number of bowel movements per 24 h until recovery (52 vs. 76 among those with a baseline of 3–5, 70 vs. 87 among those with 8 or more at baseline) were significantly smaller with racecadotril than the probiotic. The presence of specific symptoms on day 2 of treatment did not differ significantly between groups for spontaneous abdominal pain (6.86 vs. 12.42%), pain on abdominal palpation (4.57 vs. 8.70%), abdominal distension (6.29 vs. 6.21%), anorexia (10.86 vs. 7.45%), nausea (4.0 vs. 2.48%), or anal burning (1.71 vs. 2.48%). In a Kaplan–Meier analysis, the estimated probability of cure on day 2 was 42% for racecadotril vs. 21% for *S. boulardii*; respective values on day 3 were 67 and 55%; the difference between the two treatments was even more pronounced in patients with 8 or more bowel movements per day (41 vs. 11%). The incidence of adverse events was 6.8% with racecadotril vs. 7.1% with *S. boulardii*.

The final direct comparative study was performed in India and differed from most other studies in important ways (56). First, it included patients with moderate to several acute diarrhea of <5 days duration needing hospitalization, i.e., a very different patient population. Second, all patients received a base treatment of not only fluid substitution but also antibiotics (intravenous ciprofloxacin and metronidazole), again suggesting a very different study population. Because of the apparent major difference in study population and the lack of a placebo arm, we have elected not to include this trial in our analysis of placebo-controlled studies. Third, it did not use a fixed dose of racecadotril but rather defined target dose based on body weight. Fourth, it included as one of the comparator treatments the somatostatin analog octreotide, a drug which has mostly been studied in diarrhea associated with AIDS or cancer chemotherapy (16) and not approved for the treatment of acute diarrhea in any major country and, in contrast to all other comparator treatments, not available as an over-the-counter medicine requiring subcutaneous injection. This study randomized 150 patients to receive either fluid substitution plus antibiotics or additional racecadotril treatment (1.5 mg/kg) thrice daily or additional octreotide treatment (100 μg at time of hospitalization). The primary efficacy endpoint was average daily number of stools until cure. In the placebo group, this was 12.2 at time of admission, 6.8 on day 2, 3.4 on day 3, 2.1 on day 4, and 1.4 on day 5. In the racecadotril group, corresponding numbers were 12.0, 6.7, 3.4, 2.1, and 1.8 (n.s. vs. control for each time point). In the octreotide group, these numbers were 14.4, 2.3, and 1.8 on days 1, 2, and 3, respectively, with no diarrheic stools on days 4 or 5 (significant vs. control or racecadotril on day 2; Figure 4). Averaged daily quantity of stool and required volume of fluid substitution showed a similar picture. Unfortunately, the corresponding publication does not report any adverse event data.

## General Discussion

### Critique of Methods

Unfortunately, many published study reports had limited quality as judged by today’s standards. For example, some did not define a primary endpoint, did not report on randomization approach, or did not mention adverse events. Moreover, with sample sizes of 50 patients or less per study arm, some studies are likely to have had limited power to detect differences between treatments. Finally, some studies did not report statistical analysis for the comparison of racecadotril vs. comparator treatment. These limitations must be considered in the interpretation of the reported study outcomes.

Reported clinical studies with racecadotril in the treatment of acute diarrhea in adults differ in many ways. First, they have been performed in countries in Europe, Africa, Latin America, and Asia. These vary considerably in socio-economic status and health-care systems. While one of the meta-analyses comparing a Tunisian trial with those of “other countries” did not report a significant country effect (35), absence of proof is not equivalent to proof of absence. Second, allowed duration of diarrhea ranged from 3 to 5 days prior to enrollment and maximum treatment duration from 3 to 10 days. Third, actively controlled trials have involved three different comparator treatments; the treatment regimens with the comparator drug loperamide also differed between studies. Fourth, studies covered a wide range of sample sizes (31–473 per arm), yielding limited statistical power in some of them. Fifth, studies used different primary endpoints including time to cure, accumulated stool weight, number of diarrheic stools, and cumulative recovery on day 2. Finally, it is noteworthy that most studies were based on outpatients, but one included only geriatric nursing home residents (54) and one patients requiring hospital admission and receiving concomitant antibiotic treatment (56).

To better understand the efficacy and tolerability of racecadotril in the treatment of acute diarrhea in adults, two meta-analyses have recently been reported. One of them focused on efficacy and analyzed data for both the placebo and the loperamide-controlled studies (35). It did not include the placebo-controlled study from China (52) but, based on the assumption that the efficacy of *S. boulardii* remains to be proven, included the study with this probiotic as active comparator in the analysis of placebo-controlled trials. While not including an overall analysis of tolerability, it reported on rebound constipation. The second meta-analysis was overlapping with that by Vetel et al. (35) in some aspects but differed in approach from it in several other ways (34). First, similar to Vetel et al. (35), it did not include the placebo-controlled Chinese study and, in contrast to Vetel et al., did not include any of the actively controlled trials. Second, it used additional parameters for analysis of efficacy and tolerability data, including some not mentioned in the published articles describing the studies and, accordingly, not publicly accessible. Third, its main strength is an analysis based on individual patient data; this has allowed the authors to include some efficacy and tolerability parameters in the analysis that had not been reported in the corresponding publications.

These two meta-analyses (34, 35) provide important quantitative information, and this is their main strength. On the other hand, the studies included in these analyses vary considerably with regard to country of origin, allowed duration of diarrhea prior to inclusion and maximum duration of treatment, chosen comparator regimen, sample size, and reported primary outcome parameter. Therefore, we have chosen to look at each study the way it has been reported. Moreover, we have included a placebo-controlled study from China that apparently had escaped the attention of the previous analyses. We also have considered the probiotic *S. boulardii* as active comparator and, in contrast to Vetel et al. (35) not as equivalent of placebo. Finally, we have also included a study comparing racecadotril to octreotide in patients with acute diarrhea requiring hospital admission (56). Accordingly, we feel that both the meta-analysis approach and our more qualitative inspection of study data have merit and that they should be seen as complementary.

### Comparative Efficacy Outcomes

As compared to placebo, racecadotril has proven to exhibit greater efficacy. While the studies consistently showed numerically greater efficacy with racecadotril, this did not reach statistical significance with small sample sizes in an initial dose-ranging study (45). However, two meta-analyses based on aggregated data of five studies (35) or on individual patient data of four of these studies (34) clearly confirmed the overall efficacy of racecadotril as compared to placebo for the endpoint of time to cure (reported hazard ratio 1.65 [1.38; 1.97] and 1.80 [1.30; 2.50], respectively). These are consistent with the data from an additional placebo-controlled study not part of these meta-analyses (47). Of note, greater efficacy than with placebo was consistent across countries and health-care systems [France (46, 57), Tunisia (36), or China (52)] and primary study endpoints [duration of diarrhea (31), mean stool weight on first day of treatment (36), or number of diarrheic stools (46, 47)]. Taken together, these data unequivocally establish racecadotril as an effective treatment of acute diarrhea of presumed infectious origin in adults.

Seven studies have directly compared the efficacy of racecadotril to that of loperamide. While the six studies based on outpatients consistently reported similar efficacy for both treatments, that based on geriatric nursing home residents reported greater efficacy for racecadotril than for loperamide for multiple endpoints but had a relatively small sample size (54). Accordingly, the hazard ratio for better efficacy assessed as time to cure with racecadotril in the meta-analysis of all seven studies was 1.08 [0.95; 1.22] (35). The comparable efficacy of racecadotril and loperamide was consistent across slightly different treatment regimens with loperamide. Moreover, similar to the efficacy superiority relative to placebo, the efficacy equivalence relative to loperamide was consistent across countries and health-care systems and primary study endpoints. The only study reporting greater efficacy for racecadotril as compared to loperamide for duration of diarrhea also reported greater efficacy for several other efficacy endpoints including total number of diarrhea episodes, total stool output, and duration of abdominal pain (54). It also reported pharmacoeconomic superiority of racecadotril over loperamide. However, this study is difficult to interpret as it had the smallest number of patients per study arm among all trials being analyzed here, raising the possibility of random effect with small sample size. On the other hand, this study differed importantly from the others with regard to included patient populations. While other trials had recruited outpatients with a mean age of about 40 years, Galleli et al. (54) included elderly patients (range 73–96 years) in a nursing home, i.e., a particularly vulnerable population. Whether a possible efficacy benefit of racecadotril over loperamide in the elderly truly exists, requires independent confirmation of future larger trials.

One randomized study has reported the comparison of racecadotril and the probiotic *S. boulardii* (Floratil^®^) (55). While both treatments achieved “clinical success” as judged by the investigator in about 97% of patients, time to recovery and number of bowel movements per 24 h until recovery were smaller with racecadotril. Moreover, probability of cure on day 2 of treatment was twice as high with racecadotril as with the probiotic, and an even greater difference was found in the subgroup with at least eight bowel movements per day at baseline when assessed on day 3. On the other hand, individual symptoms assessed on day 2 (spontaneous abdominal pain, pain on abdominal palpation, abdominal distension, anorexia, nausea, and anal burning) did not exhibit a consistent pattern of difference; however, with overall low incidence of each symptom (<11%), the study was clearly underpowered to make such comparisons. Given the lack of reliable data on the efficacy of probiotics relative to placebo (12, 13), it is difficult to determine whether the difference in the latter two parameters reflect the superiority of racecadotril over placebo or superiority over an active comparator with weaker activity.

The study comparing racecadotril and octreotide showed greater efficacy of the latter across multiple endpoints (56). This study is the only one recruiting patients with diarrhea requiring hospitalization and antibiotic treatment, indicating that it may relate to a different patient population. Moreover, data on the efficacy and safety of octreotide in the treatment of acute diarrhea of presumed infectious origin are sparse (16) and, accordingly, octreotide has not been approved for clinical use in such patients in any major country. In contrast to all other agents covered here, it requires subcutaneous injection. Nevertheless, this study raises the intriguing possibility that octreotide may be a superior treatment of diarrhea for specific patient groups with severe illness, but the identity of such patient groups remains to be determined.

### Comparative Tolerability Outcomes

The level and granularity of tolerability reporting in studies with racecadotril in the treatment of acute diarrhea in adults has been heterogeneous. In the placebo-controlled studies, the reported adverse events were largely of mild to moderate intensity and often reflected symptoms of diarrhea such as nausea and vomiting. The incidence of adverse events was low and comparable between placebo- and racecadotril-treated patients. Accordingly, one meta-analysis reported that the risk of constipation was similar in both groups (relative risk with racecadotril 0.95 [0.24; 3.68]) with wide confidence intervals reflecting the low overall incidence (35). Therefore, both individual studies and meta-analysis data demonstrate that the tolerability of racecadotril is comparable to that of placebo.

A different picture emerged from the actively controlled studies, particularly those using loperamide as comparator. Thus, patients receiving racecadotril consistently reported a lower incidence of adverse events than those receiving loperamide. This difference was largely due to the occurrence of constipation after diarrhea had ended, although the definition of constipation differed slightly between studies. Accordingly, the meta-analysis reported the relative risk of constipation with racecadotril as compared to loperamide being 0.34 [0.22; 0.51] (35). In patients reporting constipation, its duration was also shorter with racecadotril than with loperamide treatment (49). Rebound constipation is inconvenient to the patient but perhaps even more importantly carries a medical risk due to retention of the infectious agent. In a model of newborn gnotobiotic piglets, a 4-day treatment with racecadotril (20 mg/kg b.i.d.) was associated with a similar load of *E. coli* in proximal jejunum as placebo treatment, whereas loperamide (1 mg/kg b.i.d.) treatment was associated with a much higher bacterial load (30). While the validity of this model has been challenged (58), the effect of loperamide in the gnotobiotic piglets is consistent with the effect of other opioid receptor agonists in a rat model of intestinal bacterial load (59). Moreover, in experimental studies, both in rats (racecadotril 40 mg/kg orally) and mice (racecadotril or its active metabolite thiorphan 20 mg/kg i.v.) did not affect gastrointestinal transit, whereas loperamide (2 mg/kg orally in rats, 0.5 mg/kg i.v. in mice) did (27). Accordingly, racecadotril treatment for up to 1 week did not modify oro-coecal, colonic, or overall gastrointestinal transit times (28, 29), whereas prolongation of gastrointestinal transit time and occurrence of constipation are recognized effects of loperamide (9). Therefore, the US Food and Drug Administration considers bacterial enterocolitis caused by invasive microorganisms including *Salmonella, Shigella*, and *Campylobacter* and pseudomembranous colitis associated with the use of broad-spectrum antibiotics to be a contraindication for use of loperamide (9).

Other adverse events, such as enlarged abdomen, anorexia, and abdominal pain, were also less frequent with racecadotril in a study with large sample size (50). The difference in adverse event incidence between the two groups was largest in the study in elderly patients (12 vs. 60%) (54). In contrast, the incidence of adverse events was similar and low in the comparison of racecadotril and the probiotic *S. boulardii* (55). The comparison of racecadotril and octreotide did not report adverse event data (56).

### Conclusion

In summary, racecadotril exhibited greater efficacy but similar tolerability as compared to placebo or the probiotic *S. boulardii*. In contrast, racecadotril had similar efficacy but greater tolerability, particularly less constipation and abdominal discomfort, as compared to loperamide; in an elderly population, racecadotril may have greater efficacy and better tolerability than loperamide. The occurrence of secondary constipation upon treatment of acute diarrhea is not only a matter of quality of life; rather, it may have medical consequences because of retention of infectious agent.

Apart from effects on gastrointestinal transit and constipation, other aspects also differentiate racecadotril and loperamide. Thus, loperamide metabolism involves CYP enzymes CYP 2C8 and 3A4 and is a substrate for P-glycoprotein, and inhibitors of these enzymes and transporter inhibit its elimination and increase its plasma levels upon co-administration (9). Such drug–drug interactions may also carry a risk of serious heart problems (11). In contrast, inactivation and elimination of racecadotril is not subject to such drug–drug interactions (22). Moreover, prescribing information warns to apply special caution in the use of loperamide in young children (9). Similarly, a guideline of the World Gastroenterology Association states that loperamide “is not recommended for use in children <2 years” (2). In contrast, racecadotril is registered and used in children starting at an age of 3 months.

The above limitations notwithstanding, loperamide is an effective and safe medication for the treatment of acute diarrhea of presumed infectious origin as long as the existing contraindications are observed. Therefore, loperamide is available as an over-the-counter medication in many countries. Racecadotril is also available as over-the-counter medication in many countries. As medication safety is a key criterion for the use of such non-prescription treatments, the consistently observed tolerability benefit of racecadotril over loperamide despite comparable efficacy should favor its use, particularly as over-the-counter medication.

While the above studies clearly define a role for racecadotril in the treatment of acute diarrhea in adults relative to placebo or to loperamide, they also point to three relevant directions for future research in this area. First, the study reporting a much greater efficacy with racecadotril as compared to loperamide is the only one performed in a geriatric population in a nursing home setting (54). However, it also had the smallest sample size among the studies discussed here. As the elderly are particularly vulnerable to diarrhea-induced dehydration, additional data in elderly populations are required to derive treatment recommendations in this patient group. Second, probiotics are a guideline-recommended treatment for acute diarrhea, particularly in children, these recommendations have been criticized because of limited strength of the underlying data with regard to methodology and sample sizes (12–14). Only with more clarity on the value of probiotics in the treatment of acute diarrhea in adults, one can place the reported benefit of racecadotril over *S. boulardii* (55) in perspective. Finally, one report has suggested that octreotide may be more effective than racecadotril (56) but is based on a patient population differing considerably from those in all other studies. Specifically, Mehta et al. (56) have studied patients with such severe diarrhea that hospitalization and concomitant antibiotic treatment were required. Moreover, that study unfortunately has not reported on adverse events observed with the two treatments. Additional studies will be required to define the benefit/risk ratios of octreotide and racecadotril in patients with severe diarrhea requiring hospitalization.

## Author Contributions

WF, VA, TM and PL have substantially contributed to the development of the manuscript outline and literature search strategy, have critically reviewed manuscript drafts for intellectual content, have approved the version to be published, and agreed to be accountable for all aspects of the work in ensuring that questions related to the accuracy or integrity of any part of the work are appropriately investigated and resolved. ME substantially contributed to the development of the manuscript outline, has led the literature search, has critically reviewed manuscript drafts for intellectual content, has approved the version to be published, and agreed to be accountable for all aspects of the work in ensuring that questions related to the accuracy or integrity of any part of the work are appropriately investigated and resolved.

## Conflict of Interest Statement

Dr. ME and Dr. TM are employees of Boehringer Ingelheim Pharma GmbH & Co KG. The remaining authors declare that the research was conducted in the absence of any commercial or financial relationships that could be construed as a potential conflict of interest.
